# The Psychotomimetic Nature of Dreams: An Experimental Study

**DOI:** 10.1155/2012/872307

**Published:** 2012-03-26

**Authors:** Oliver Mason, Dominic Wakerley

**Affiliations:** Research Department of Clinical, Educational and Health Psychology, 1-19 Torrington Place, University College London, London WC1N 6BT, UK

## Abstract

Several theories promote the similarities between dreaming and psychosis, but this has rarely been tested empirically. We assessed dreaming and waking reality using the Psychotomimetic States Inventory, a measure of psychotic-like experience originally designed for drug studies. Twenty participants completed the measure in each of two dream conditions and one waking condition. Dreams were assessed upon waking naturally and also using a movement-activated (actigraph) alarm during the night. Overall, participants reported more quasipsychotic characteristics during dreams (in both conditions) than when awake. This was most marked for paranoia and delusional thinking, but differences were also seen for perceptual abnormalities, mania, and anhedonia. The quality of dream experience seems particularly similar to psychosis in sometimes being highly self-referential and having a paranoid content. Subjective changes to cognition and affect are consistent with alterations in prefrontal cortical activity during REM sleep that mirror those of schizophrenia.

## 1. Introduction and Methods

The phenomenological similarity between the dream state and psychosis has been often remarked upon but rarely empirically tested. Kant declared “the lunatic is a wakeful dreamer,” while Schopenhauer said “a dream is a short-lasting psychosis, and a psychosis is a long-lasting dream.” Perhaps the most developed neurobiological account, Hobson [1] has advanced a recent hypothesis of the dreaming brain as a model of psychosis, suggesting dreams mimic the distortion of reality seen in the positive symptoms of schizophrenia, though he prefers the term “delirium.” “In dreams, as in delirium, consciousness is clouded, attention is distractible, intellectual functions are dull, perceptions are hallucinatory, cognition is illogical, emotion is unstable and uncontrolled, memory is poor, and thought processes are at a concrete, not a symbolic, level” [2, page 23]. Although in some ways adversaries of Hobson's wider model (see [3]), Sohms and Turnbull also advocate that “the functional anatomy of dreaming is almost identical to that of schizophrenic psychosis” [4, page 213]. Pushing the analogy to the furthest by describing psychosis itself as a dream state with the neurobiology of REM stage sleep, Gottesmann [5] outlines the best delineated areas of neurobiological overlap in terms of “common intracerebral disconnections, disturbed responsiveness and sensory deafferentation processes (and) dorsolateral prefrontal deactivation” (page 1105). 

The commonest metric for assessing and asserting this commonality has been that of dream bizarreness, though the frequency of bizarre elements seems to vary depending on what is included, and may well be overclaimed by some theorists [3]. One large study of dream recall [6] found 36% to have more than one bizarre element, while another found bizarreness in 14% of dream characters [7]. If small distortions are included, figures rise to 40–60% (e.g., [8]). As Domhoff (page 4, [3]) notes “it is not obvious that […] unusual events in dreams are inherently bizarre if they are compared with either the stories people are familiar with in waking life or waking thought flow.” Interestingly, schizophrenia patients' waking fantasies may be more like dreams at least as measured by the Thematic Apperception Test rated for cognitive bizarreness [9].

Whether dreams take the form and content of types of delusional and hallucinatory experiences reported by patients is an interesting aspect of the question: two recent studies by Yu [10, 11] have attempted to characterize typical dreams in order to elucidate a typology of what is recalled of these. Paranoia and grandiosity were repeated factors that shared much with common delusional themes, while other more ego-ideal contents did not.

### 1.1. Aims and Hypotheses

A naturalistic setting is crucial to any study of dream content, as it has been repeatedly found that in dream laboratories the sleeper dreams of dream laboratories: thus we aimed to capture REM-sleep dreaming by the least intrusive means possible. In addition to testing upon waking naturally, the study used an activity-monitoring wristwatch set to wake the participant when movement (potentially REM-related) was detected. As major body movements occur predominantly before and after REM sleep periods [12], we hypothesized that use of the actigraphically triggered alarm would maximize dream recall. Actigraph-based methods have a good degree of correspondence with the gold standard of polysomnography for sleep recording in good sleepers at least [13, 14]. To measure the psychotic qualities of dreams, we used the Psychotomimetic State Inventory (PSI, [15]), a measure developed for recreational drug studies and other analogous settings (e.g., sensory deprivation [16]). This is not a symptomatic measure; rather it assesses schizotypal experiences that are thought to be analogous to psychosis. We hypothesized that dreams would be characterized by greater PSI scores that the natural awake state. In particular, we hypothesized greater paranoia and delusional content based on the earlier work of Yu [10, 11].

### 1.2. Method

20 participants (13 male and 7 female) with an average age of 20.5 took part. All were undergraduate students with no history of psychiatric or neurological disorder. In this within-subject repeated measures design, all were asked to complete the PSI on three occasions: upon waking naturally; following artificial waking using actigraph recording; in the normal waking state during the day. Participants recorded their responses in their own homes following thorough briefing on the use of the questionnaires and the actigraph watch (Sleep Tracker Pro, Innovative Sleep Solutions Inc., Atlanta, GA, USA). As REM sleep stages last longer towards the end of the sleep cycle, the watch alarm was set to respond to movement within a two-hour period around an hour prior to natural waking for each participant. Participants were asked to refrain from use of alcohol or recreational drugs on the day prior to testing. As use of the actigraph watch necessarily required curtailing sleep this was only performed on a single night; however, participants were able to report on a dream upon natural waking should this occur while in the study over a one week period. Waking state data was obtained by sending a text message reminder at time of day, 6 hours after the individual's usual stated waking time (most usually in the early afternoon). University College London ethical approval was obtained.

#### 1.2.1. Psychotomimetic State Inventory [13]

Participants rate statements that describe their experience from 0 (not at all) to 3 (strongly) across 48 items: the instructions state “Please complete the following questions by circling the number that best describes your experience *in the past few hours*.” The PSI yields six subscales: “delusional thinking,” “perceptual distortion,” “anhedonia,” “mania,” “paranoia,” and “cognitive disorganization.” The scale has a test-retest reliability of 0.84 and a Cronbach's alpha overall of 0.94.

## 2. Results

One participant failed to recall a dream by either method, one more after waking naturally over the course of the week and two further participants did not recall a dream using the actigraph. 

**Figure 1:**
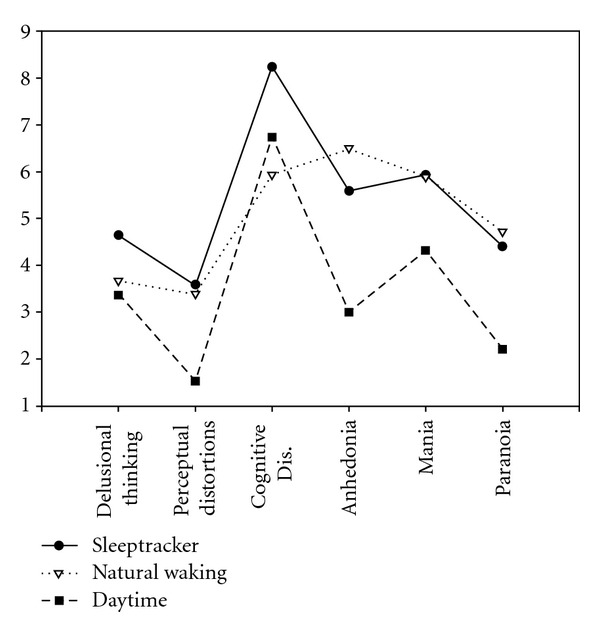
PSI results across the three conditions.

Results using the PSI in the three conditions (awake, dream-natural waking, and dream-sleeptracker waking) are given in Table 1 and illustrated in Figure 1. Repeated measure analyses of variance were conducted to test the hypotheses (see Table 1 for planned comparisons). Overall, both dream conditions produced greater overall PSI scores than those reported during the day. Dreams were rated as having greater psychotomimetic qualities than the daytime-awake state, with no difference between those reported when waking naturally and those using the sleeptracker device, though there were slightly greater ratings for the device. Dream/awake differences were seen for almost all subscales except for cognitive disorganization. Although some dream/awake differences failed to reach significance, in every case the pattern was the same with, both dream conditions tending to have higher means than that of the daytime-awake condition. In line with the hypotheses, this was most marked for delusional thinking and paranoia. However, significant differences were also seen for anhedonia and mania suggesting changes in affect are also important aspects of dream experience that parallel psychotic experience. 

## 3. Discussion

The study attempted to measure dream experience as soon as possible after it occurred in addition to test completion upon waking. The use of an actigraphic approach, waking participants during periods of greater movement during sleep did successfully elicit dream material for seventeen of the twenty participants. We would, therefore, tentatively suggest the utility of actigraphy in obtaining dream reports, though their sleep staging cannot be directly ascertained.

The hypothesis that dreams would be characterized by greater PSI scores than the “daytime-awake” state was confirmed both for the dreams recalled using actigraphy and upon natural morning wakening. Dreams were characterized by significantly greater delusional thinking, perceptual distortions, anhedonia, mania, and paranoia. Thus, the results are consistent with those of Yu [10, 11] on delusional content and paranoid ideation. In the present study, many of the items concern ideas of reference or special/magical powers; the scores suggest a high degree of self-referential dream content. Paranoia was the largest effect of all and suggests common experiences of being watched, plotted against, or harmed with associated fear. This is consistent with one evolutionary theory that dreams are specialized to simulate threatening events [8].

Differences in mania and anhedonia were perhaps more surprising and suggest cognitive-affective experience is altered in additional ways that resemble psychotic experience. Perusal of the anhedonia questions, many of which tap feeling distant, uninvolved or uncomfortable with people, suggest that in dreams, one's experience of self in relation to others is in some ways altered, or experienced at one stage removed. Whether this is a true parallel to anhedonia in the psychotic sense is an intriguing idea as many neurocognitive accounts of negative symptoms focus on failures to construct and sustain accurate representations of oneself and others—itself a fair description of some dreams.

 Mania taps a sense of increased subjective speed of thought and action, mood swings, and greater impulsivity. A weakened sense of cognitive control is a clear parallel between the dream and psychotic states. The organization of cognitive control within the lateral prefrontal cortex (LFFC) in both normals and schizophrenia patients is only now being delineated by fMRI ([17]). Schizophrenic patients exhibited *hypoactivation* in caudal LPFC regions suggesting a weakening of contextual influence and thus inappropriate behavioural representation (and hence bizarre behaviour). They interpreted *hyperactivation* in rostral LPFC regions as an attempt to compensate by drawing excessively on temporal episodic information. While the caudal LPFC may or may not be deactivated in NREM sleep, it certainly is in REM, and other areas of PFC are activated including orbital frontal areas that serve temporal and emotional processing (see [18] for review). However, complex patterns of specific landscapes of activation are associated with different sleep stages, and it is difficult to allocate specific changes to dreaming or individual aspects of dream experience: as a recent review [19, page 1004] states “no study to date has been able to directly relate, in the same individuals, brain activation during sleep with emotions experienced in dreams.” We are simply drawing the analogy between the heightened sense of emotion reported in some dreams and clinical states of mania or paranoia. Of course a wide range of emotions occur in dreams with fear and anxiety prominent among these: Schwartz and Maquet [20, page 25] highlight the role of the amygdala in organizing emotional processing during sleep in a way that “might favor fearful experience in dreams.” Even amongst a range of structural and functional alternations seen in limbic structures in schizophrenia, grey matter loss in the left amygdale appears particularly marked and extends to patients' relatives [21].

 It is important to note several limitations. The study does not compare dreaming with schizotypal experiences occurring when awake, and indeed there may be bias introduced by the methods of obtaining dream experience ratings as compared to waking state ratings. In retrospect, averaging across a large set of data points would help provide a richer picture of whether dreams differ from schizotypal mentation when awake. As a measure derived from psychotic phenomena was used, assessment of visual hallucinosis formed a very small aspect of the measurement (two items of 48). As a consequence, the study was very definitely *not* a test of the “dream-as-psychosis” versus “dream-as-delirium” hypotheses. The final limitation is that sleep-staging was not directly assessed, and no neurobiological correlates of dream experiences obtained.

## Figures and Tables

**Table 1:** Means and standard deviations of PSI and subscales.

	Dream-natural waking (NW)	Dream-sleeptracker (ST)	Awake-daytime (AD)	Pairwise comparisons
PSI Total**	27.3 (16.9)	33.1 (21.8)	18.5 (14.2)	NW = ST > AD
Delusional thinking**	3.2 (2.9)	5.9 (5.4)	2.1 (2.3)	ST > AD
Perceptual distortions*	3.4 (4.1)	3.6 (4.3)	1.5 (2.1)	NW = ST > AD
Cognitive Disorganization	5.9 (5.1)	8.2 (6.4)	6.7 (5.3)	—
Anhedonia*	6.5 (3.2)	6.0 (3.8)	3.0 (3.0)	NW > AD
Mania*	5.9 (3.8)	5.9 (2.9)	4.3 (3.1)	NW = ST > AD
Paranoia**	4.7 (4.4)	4.4 (4.7)	1.4 (1.8)	NW=ST>AD

*P < .05, **P < .01 RM-ANOVA, and pairwise comparisons *P* < .05 Bonferroni corrected.
